# Identification of MTHFD2 as a prognostic biomarker and ferroptosis regulator in triple-negative breast cancer

**DOI:** 10.3389/fonc.2023.1098357

**Published:** 2023-01-16

**Authors:** Hao Zhang, Shuangli Zhu, Haiting Zhou, Rui Li, Xiaohui Xia, Huihua Xiong

**Affiliations:** Department of Oncology, Tongji Hospital, Tongji Medical College, Huazhong University of Science and Technology, Wuhan, Hubei, China

**Keywords:** MTHFD2, breast cancer, bioinformatics, prognosis, biomarker, ferroptosis

## Abstract

**Background:**

Methylenetetrahydrofolate dehydrogenase 2 (MTHFD2) is a mitochondrial bifunctional enzyme encoded in the nucleus. It plays a significant role in the regulation of glucose, nucleic acid, and folate metabolism, and maintains redox balance in the cells. The present study aimed at elucidating the potential function and mechanisms of MTHFD2 and explored the correlation between ferroptosis and MTHFD2 in triple-negative breast cancer.

**Methods:**

MTHFD2 expression, survival analysis, and clinical correlation were performed using data from various online databases including TCGA, GEO, HPA, GTEX, Kaplan–Meier Plotter, PrognoScan, and UALCAN databases. Genomic alterations and CNV analysis were performed using the cBioPortal and GSCA databases. Potential functions and mechanisms were explored by enrichment analysis. The tumor microenvironment was identified by the TIMER database. *In vitro*, RT-qPCR and western blot assays were utilized to identify the MTHFD2 expression and the knockdown effects in breast cancer. CCK8, cell wound healing, transwell, and flow cytometry assays were used to identify the potential function of MTHFD2 in TNBC cells. MDA, GSH detection, and flow cytometry assays were performed to identify ferroptosis. Western blot assays were performed to measure the protein expression of all target genes.

**Results:**

MTHFD2 expression levels were up-regulated in the majority of cancers and particularly in TNBC, in which higher expression levels indicated a poorer prognosis. Enrichment analyses showed that MTHFD2 is involved in various tumor-related biological processes. MTHFD2 expression was found to strongly correlate with multiple immune cell infiltration. *In vitro*, the knockdown of MTHFD2 suppresses the proliferation, apoptosis, migration, and invasion in TNBC cells. In addition, the MTHFD2 knockdown significantly enhanced intracellular ROS and lipid peroxidation and decreased intracellular GSH. The expressions of SLC7A11, GPX4, and NRF2 were down-regulated by the MTHFD2 knockdown.

**Conclusion:**

MTHFD2 could be a crucial molecular biomarker for predicting patient prognosis and a novel therapeutic target in TNBC. In addition, MTHFD2 is a potential ferroptosis regulatory gene in TNBC.

## Introduction

Breast cancer is the commonest cancer worldwide and causes a considerable number of tumor-related deaths, seriously threatening public life and health (1). Basal, Luminal A, Luminal B, and HER2 amplification subtypes were the major subtypes in breast cancer, which were classified based on the expression condition of Ki67, estrogen receptor (ER), and progesterone receptor (PR), human epidermal growth factor receptor 2 (HER-2) (2). Triple-negative breast cancer (TNBC) is a specific type of basal subtype, characterized by the absence of ER, PR, and HER-2, accounting for approximately 15% to 20% of all breast cancer. Patients with TNBC usually demonstrate early metastasis, high rates of recurrence, and drug resistance due to poor differentiation and highly malignant biological characteristics, which leads to a worse prognosis (3). As validated therapeutic targets are lacking in TNBC, traditional treatments including surgery, radiotherapy, and chemotherapy remain the mainstay of treatment (4). Consequently, it is essential to develop new therapeutic targets and strategies for its management.

MTHFD2 is an important mitochondrial protein, which is encoded by the nucleus and is involved in a variety of cellular metabolic processes (5). It participates in nicotinamide adenine dinucleotide phosphate (NADP^+^)-dependent folate metabolism and catalyzes the generation of CHO-THF by the dehydrogenation of CH2-THF (6). The important one-carbon unit compound produced during the metabolism of folate is involved in the synthesis of purine and thymidylic acid (7, 8), providing the basic conditions for the synthesis of nucleic acid. Owing to its NADP^+^-dependent metabolic enzyme characteristics, MTHFD2 can affect the redox balance in cells by changing NADPH/NADH levels during intracellular metabolism (9). Ju et al. found that inhibition of MTHFD2 could suppress the proliferative and invasive ability of tumor cells by reducing intracellular NADPH and disturbing redox homeostasis (10, 11). There is increasing evidence to suggest that MTHFD2 expression is relevant to tumor progression in many cancers, including head and neck cancer (12), lung cancer (13), kidney cancer (14), and ovarian cancer (15). MTHFD2 expression levels have been reported to be higher in human embryonic cells, while most normal adult tissues demonstrate low expression (16, 17), which provided a theoretical basis for reliably targeting MTHFD2 for the treatment of cancer.

The tumor microenvironment (TME) is composed of non-cancerous cells and cell-secreted components, among which the cellular components mainly include fibroblasts and endothelial and immune cells, and the non-cellular components are mainly chemokines, cytokines, growth factors, and extracellular vesicles (18). As immune cells in the TME recognize and kill tumors, their function and regulatory mechanisms are one of the most widely studied areas in the field of anti-cancer research (19). The interaction between tumors and immune cells plays a significant role in regulating the occurrence, progression, and therapeutic outcomes of tumors (20).

Ferroptosis is a specially programmed cell death (RCD) process different from apoptosis and pyroptosis, which is dependent on iron and caused by lipid peroxide overload on the cell membrane. It is characterized by iron-catalyzed peroxidation of polyunsaturated fatty acid containing phospholipids and increased intracellular reactive oxygen species (ROS) (21). As a novel mechanism for the regulation of cell death, ferroptosis has gained considerable attention in the field of tumor biology and therapeutics research in recent years. Numerous studies have demonstrated that several genes and signaling pathways which participate in the regulation of tumor physiological processes are also involved in ferroptosis regulation (22, 23). Results from various experiments have also demonstrated that ferroptosis may enhance the efficacy of anti-cancer therapies including radiotherapy, immunotherapy, chemotherapy, and targeted therapy (24, 25). However, the function and regulatory mechanisms of ferroptosis and the relationship between MTHFD2 and ferroptosis in TNBC cells remain unclear and need further investigation.

In the present study, we employed a bioinformatics approach to evaluate MTHFD2 expression levels in various tumors and investigate its relationship with patient prognosis. We then explored genomic alterations and biologically relevant functions of MTHFD2 in breast cancer. We also assessed the correlation between MTHFD2 expression and immune cell infiltration in the TME of breast cancer. Finally, we verified the function of MTHFD2 in TNBC *via in vitro* experiments based on data from bioinformatics predictions. We demonstrated a regulatory association between MTHFD2 and ferroptosis and identified the specific mechanisms for this association. The presentation aimed to identify the biological function and potential regulatory mechanisms of MTHFD2 and provide a novel approach for enhancing therapeutic effects in TNBC.

## Materials and methods

### Data source

The pan-cancer and breast cancer-related gene expression RNAseq and survival data were obtained from the Cancer Genome Atlas (TCGA) database and downloaded from the University of California Santa Cruz Xena website. RNA sequencing data for human normal tissue gene expression were obtained from the Genotype Tissue-Expression portal. The data from the GSE22820, GSE109169, GSE45827, GSE25066, and GSE20685 datasets were obtained from the Gene Expression Omnibus (GEO). The web addresses of all databases, online websites, and online analysis tools have been provided in Supplementary Table S1.

### Expression level analysis

The expression and survival date of MTHFD2 were extracted from TCGA, GTEX, and GEO databases. All of the data was analyzed by a bioinformatics online analysis website based on the R studio 3.6.3. The UALCAN database based on TCGA was used to identify differential expression of MTHFD2 in various breast cancer subtypes. Immunohistochemistry staining data were obtained from the Human Protein Atlas (HPA) database to explore MTHFD2 protein expression patterns in tumor and normal breast tissue.

### Survival analysis

​Kaplan-Meier, nomogram, and ROC curves were plotted by the online analysis website based on the date of the TCGA data. The Kaplan–Meier Plotter database and TISIDB database were used to plot the Kaplan-Meier curves for evaluating the value of MTHFD2 mRNA expression in breast cancers. The GSE12276, GSE9893, and GSE3494 datasets were obtained to plot the breast cancer survival curves.

### Genomic alterations and CNV analysis

​ Data regarding cancers with mutations in the *MTHFD2* gene were analyzed using the cBioPortal database. Data about copy number variations (CNV) of MTHFD2 in various cancers were obtained from the Gene Set Cancer Analysis (GSCA) website.

### Enrichment analysis

Gene Set Enrichment Analysis (GSEA) was performed using GSEA software version 4.1.0. The data were obtained from the TCGA database, |NES| >1, FDR <0.25, and P.adj <0.05 were considered to be statistically significant. Kyoto Encyclopedia of Genes and Genomes (KEGG) and Gene Ontology (GO) enrichment analyses were used to investigate MTHFD2-related functions. All analyses were conducted through the online analysis website.

#### Immunology correlation analysis

The association between MTHFD2expression conditions and the major immune cells subtype was conducted by the TI SIDB database. The association between the MTHFD2 expression levels and multiple immune cell infiltration, estimate score, stromal score, and immune score in breast cancer were analyzed by the online analysis website. The association between the MTHFD2 mutation situations and immune cell infiltration was explored by using the TIMER database. The association between the MTHFD2 expression and immune-related molecular biomarkers was explored by the TIMER and TCGA databases.

### Cell culture

Human breast cancer cell lines (including BT474, MDA-MB-468, MCF7, SKBR3, and MDA-MB-231) and the human normal breast cell line (MCF10A) used in this study were maintained at the Tongji Hospital Laboratory, Department of Oncology (Wuhan, China). All cell lines were purchased from the American Type Culture Collection (ATCC). The cancer cells were cultivated in RPMI 1640 or Dulbecco’s Modified Eagle Medium. MCF 10A cells were cultivated in a specific medium, which was purchased from Priscilla (Wuhan, China). The cells were incubated with 10% fetal bovine serum (FBS) in 5% carbon dioxide at 37°C.

### RNA interference

The RNA interference lentiviral vectors were structured by Genechem (Shanghai, China). The interference sequence is as follows, shRNA#1: GCCTCTTCCAGAGCATATTGA, shRNA#2: GCAGTCATTGATGTGGGAATA. The negative control (NC) sequence is TTCTCCGAACGTGTCACGT. All operations were performed as per the user manual of Genechem.

### RT–qPCR

Trizol (Servicebio, Wuhan, China) reagent was utilized to extract all cell RNA. The extracted RNA was conducted to synthesize cDNA by reverse transcription using HiScript II Q RT SuperMix (Vazyme Biotech, Nanjing, China) reagent. Then, RT–qPCR was performed with ChamQ Universal SYBR qPCR Master Mix (Vazyme Biotech, Nanjing, China) reagent. The genes primer sequences mentioned are available in **Supplementary Table S2**.

### Cell viability assay

Cell Counting Kit‐8 assay (CCK-8) was performed to assess cell viability and the appropriate number of cells were selected for seeding into 96-well plates. CCK-8 reagents (MCE, HY-K0301, USA) were dissolved in a serum-free medium at a concentration of 10% and added to each well at a predefined time. The absorbance at 450nm (OD450) was recorded after incubation for approximately 2 hours. Z-VAD-FMK (MCE, HY-16658B, USA) and Ferrostatin-1 (MCE, HY-100579, USA) were dissolved in dimethyl sulfoxide (DMSO) and diluted to concentrations of 20 µm/L and 1 µm/L, respectively, using the complete medium. Z-VAD-FMK and ferrostatin-1 were added to the 96-well plates with the adherent cells. Cell viability was then detected at the appropriate time using the CCK-8 assay.

### Wound healing assay

The appropriate number of cells was plated in 12-well plates in advance. After attaining sufficient confluence on the plate, straight lines were scratched onto the monolayer cells using an appropriate pipette tip. Next, phosphate-buffered saline was utilized to wash the scratched cells. Then, they were cultivated with a moderate FBS-free medium. The wound healing status was observed and recorded under a microscope at 0h, 24h, and 48h after scratching.

### Transwell assay

Migration and invasion capabilities were evaluated using transwell assays in a 24-well plate. For the invasion assay, the matrigel matrix (BD Science, MD, USA) was spread over the upper chamber in advance. The appropriate number of cells were suspended in 100 µl FBS-free medium and planted in a transwell chamber, and a 500ul medium containing 15% FBS was added to the 24-well plate. After incubation for 24 h, the chambers were rinsed with phosphate-buffered saline and fixed with formaldehyde for approximately 20 min. Finally, the cells were stained by placing them in 0.5% crystal violet for approximately 20 min. The migration and invasion status were evaluated under a microscope.

### Malondialdehyde and glutathione content analysis

Commercially available detection kits were used to analyze levels of malondialdehyde (MDA) (Solarbio, Beijing, China) and glutathione (GSH) (Jiancheng Bioengineering, Nanjing, China) in TNBC cells. The gray values were measured using the microplate reader. All procedures were performed according to the manufacturer’s instructions.

### Western blot analysis

All the cell proteins were extracted by RIPA (Servicebio, Wuhan, China) with PMSF (Servicebio, Wuhan, China) and phosphorylase inhibitors (Servicebio, Wuhan, China). BCA (Promoter, Wuhan, China) reagent was utilized to evaluate the concentration of samples. 30ug of proteins were loaded onto the sample lane for 10% SDS polyacrylamide gel electrophoresis (SDS-PAGE) and then transferred onto the PVDF membrane (Millipore, USA). The membranes containing proteins were blocked with 5% BSA solution for 1h and incubated overnight on the shaker with targeting protein antibodies at 4°C. After washing twice with Tris-buffered Saline with Tween, the membrane-containing proteins were incubated on the shaker with the secondary antibody for approximately 1 h. The targeted proteins were finally developed using an exposure machine. Data regarding the antibodies have been provided in **Supplementary Table S3**.

### Flow cytometry

The PE Annexin V Kit (BD, 559763, USA) was utilized to measure the apoptosis status of the cells. The tested cells were collected into centrifuge tubes and incubated with 7AAD and Annexin V dye for 15 min at 25°C. The prepared cells were identified by flow cytometry machine (Beckman, USA) with PE and PerCP fluorescence channel. Reactive Oxygen Species (ROS) detection kit (Beyotime, S0033S, China) was utilized to measure ROS in the cells. The BODIPY 581/591 C11 probe (Invitrogen, D3861, USA) was used to measure intracellular lipid peroxidation. The cells were incubated for 30 min after adding the probe. A flow cytometry machine with a FITC channel was used for evaluation. All results were analyzed using FlowJo software version 10.8.1.

### Statistical analysis

All of the statistical graphs and statistical results were analyzed using GraphPad Prism 9.0 software. Kaplan–Meier survival curses were performed to evaluate the survival differences. The Spearman correlation coefficient was utilized for the correlation analysis. The t-test and the one-way analysis of variance (ANOVA) were conducted to compare experimental data from two groups and multiple groups, respectively. All data have been presented as means ± SD, *p <*0.05 was considered statistically significant (ns: *p*≥0.05, *: *p*<0.05, **: *p*<0.01, ***: *p*<0.001, and ****: *p*<0.0001).

## Results

### The expression level and patients’ survival effect of MTHFD2

**Figure 1:**
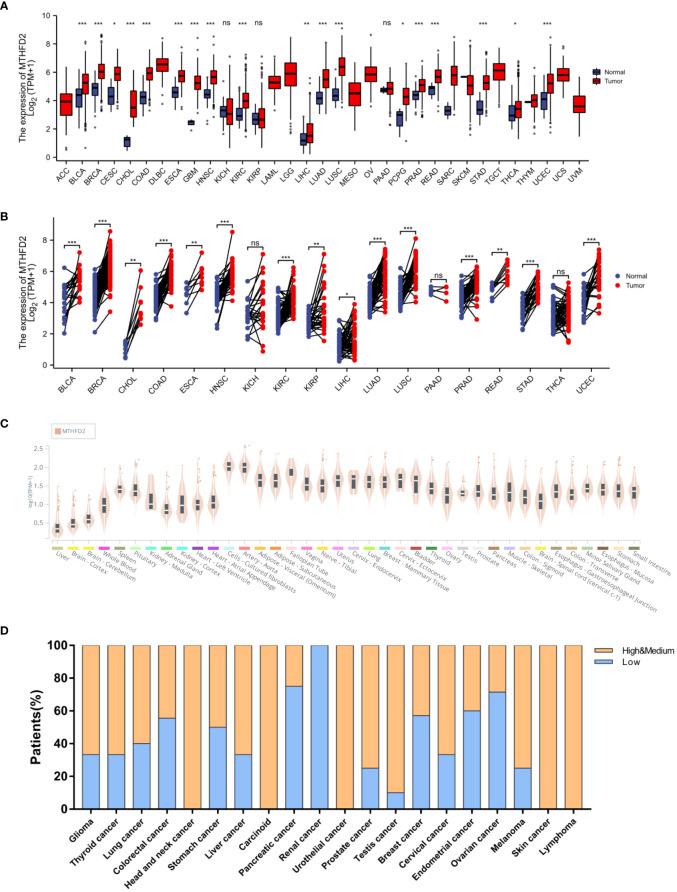
MTHFD2 expression in various normal and tumor tissues. Differential MTHFD2 mRNA expression levels in various normal tissues and tumors. **(A)** On unpaired sample analysis and **(B)** On paired sample analysis. Red represents tumor tissues and blue represents normal tissues. **(C)** MTHFD2 mRNA expression levels in normal tissues. **(D)** Protein expression pattern of MTHFD2 in various cancers. Orange indicates high or moderate MTHFD2 protein expression, while blue indicates low MTHFD2 protein expression. ns: *p*≥0.05, *p<0.05, **p<0.01, ***p<0.001, and ****p<0.0001.

On unpaired and paired analysis, we found the expression levels of MTHFD2 to be increased in the majority of tumor tissues in contrast to normal tissues (**Figures 1A, B**). We then investigated the MTHFD2 mRNA expression status in human normal tissues from the Genotype Tissue-Expression database. Our results confirmed MTHFD2 expression levels to be generally low in the majority of human tissues except for fibroblasts and aortic tissues (**Figure 1C**). We also investigated MTHFD2 expression in various tumors. The results demonstrated moderate or high levels of MTHFD2 protein expression in most cancers except for those of the pancreas, kidney, and ovary (**Figure 1D**).

**Figure 2:**
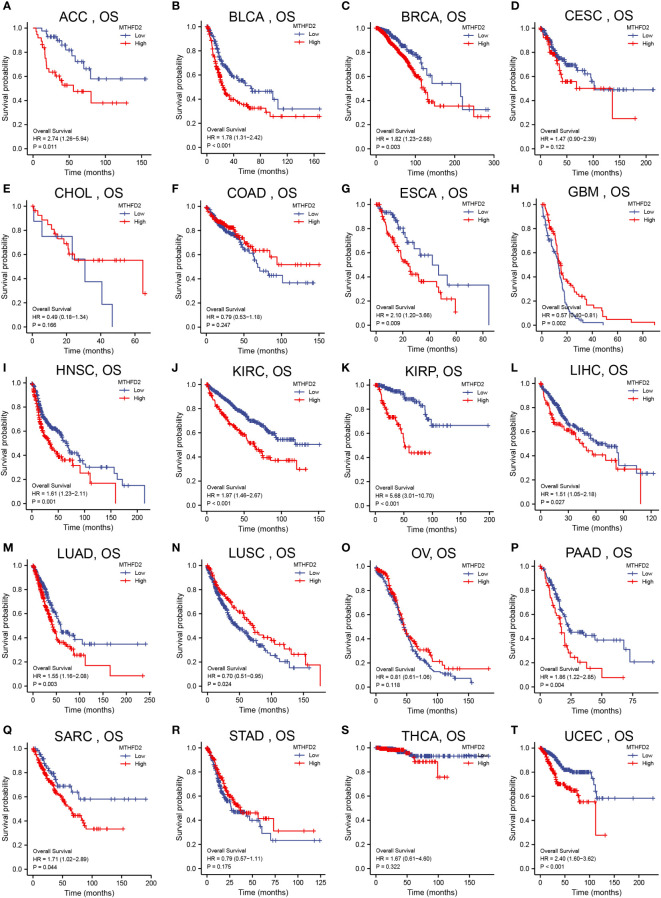
Pan-cancer patient survival analysis. Overall survival (OS) analysis of MTHFD2 in different tumors using Kaplan–Meier survival curves. **(A)** ACC, **(B)** BLCA, **(C)** BRCA, **(D)** CESC, **(E)** CHOL, **(F)** COAD, **(G)** ESCA, **(H)** GBM, **(I)** HNSC, **(J)** KIRC, **(K)** KIRP, **(L)** LIHC, **(M)** LUAD, **(N)** LUSC, **(O)** OV, **(P)** PAAD, **(Q)** SARC, **(R)** STAD, **(S)** THCA, **(T)** UCEC. Red lines indicate high MTHFD2 expression, while blue lines indicate low MTHFD2 expression.

We had investigated the relationship between patient overall survival (OS) and MTHFD2 expression status using Kaplan-Meier curves. The results showed that high MTHFD2 expression levels were usually indicative of a poorer prognosis in most cancer types except for cervical squamous cell carcinoma and endocervical adenocarcinoma, cholangiocarcinoma, colon adenocarcinoma, glioblastoma multiforme, lung squamous cell carcinoma, ovarian cancer, stomach adenocarcinoma, and thyroid carcinoma (**Figure 2**).

### Genetic alteration analysis

The genomic instability is a significant characteristic of tumorigenesis, malignant progression, distant metastasis and treatment-resistant (26). The genetic alteration in cancer cells directly or indirectly leads to the development of tumors (27). The mutation of p53 damages cellular DNA repair, and cell cycle arrest and promotes apoptosis in injured cells (28).

**Figure 3:**
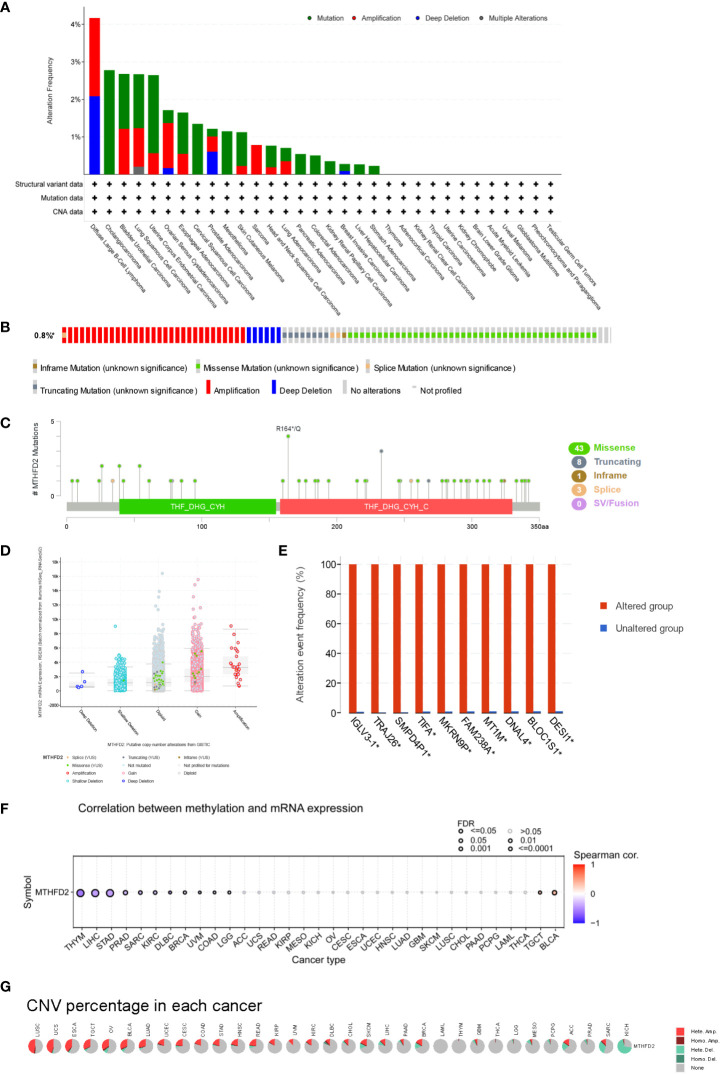
Genetic Alteration of MTHFD2. **(A)** Genetic alterations of MTHFD2 in various tumors. **(B)** Summary of structural variations, mutations, and copy number alterations of MTHFD2. **(C)** Summary of mutation types, number, and sites of MTHFD2 gene alterations. **(D)** Specific change type of MTHFD2. **(E)** The frequency of top 10 gene variations between the MTHFD2 abnormal and normal groups. **(F)** Correlation analysis between DNA methylation and MTHFD2 mRNA expression. **(G)** Assessment of MTHFD2 CNV alterations in cancers.

MTHFD2 genetic alteration analysis showed mutations to be the dominant type of alteration, followed by amplifications. In this context, bladder urothelial carcinoma, large B-cell lymphoma, diffuse uterine corpus endometrial carcinoma, lung squamous cell carcinoma, and cholangiocarcinoma were the primary tumor types with higher MTHFD genetic alterations (> 2%) (**Figure 3A**). The main genetic alterations of MTHFD2 included amplification, deep deletion, and multiple types of mutations (**Figure 3B**). The number of cases with MTHFD2 gene modifications among specific sites and types were also investigated (**Figure 3C**). The findings suggested that missense mutations were the predominant type of alteration. Amplification, gain-of-function, and diploidy were the most common copy number alterations in MTHFD2 (**Figure 3D**). In the MTHFD2 group with alterations, *IGLV3-1*, *TRAJ26*, *SMPD4P1*, *TIFA*, *MKRN9P*, *FAM238A*, *MT1M*, *DNAL4*, *BLOC1S1*, and *DESI1* genes were identified as the top 10 genes with the highest alteration frequency (**Figure 3E**).

Analysis of the correlation between MTHFD2 expression and DNA methylation showed that methylation significantly suppressed MTHFD2 expression in THYM, LIHC, and STAD (**Figure 3F**). The percentage and contribution of CNV to pan-cancer MTHFD2 expression were examined. The findings demonstrated a higher percentage of CNV in LUSC, UCS, ESCA, TGCT, OV, BLCA, and LUAD. Heterozygous amplification accounted for the largest proportion of CNVs (**Figure 3G**).

### The MTHFD2 expression status and its clinical significance in breast cancer

**Figure 4:**
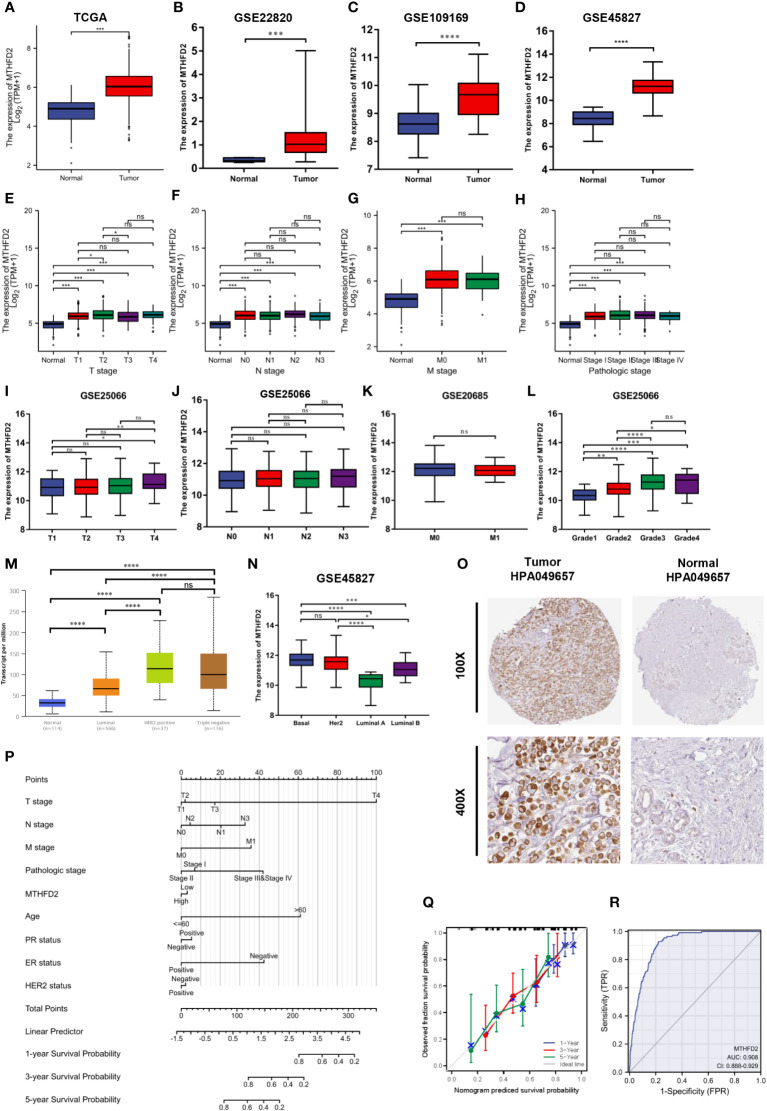
MTHFD2 expression status and clinical significance. **(A–D)** MTHFD2 expression level in breast cancer and normal tissues. **(A)** TCGA-BRCA, **(B)** GSE22820, **(C)** GSE109169, **(D)** GSE45827. **(E–H)** Relationship between MTHFD2 expression and clinical features. **(E)** T stage, **(F)** N stage, **(G)** M stage, **(H)** pathologic stage. **(I–L)** The relationship between MTHFD2 expression and clinical features from the GEO database. **(I)** T stage from GSE25066, **(J)** N stage from GSE25066, **(K)** M stage from GSE20685, **(L)** Grade from GSE25066. **(M, N)** Relationship between MTHFD2 mRNA expression and molecular subtype of breast cancer. **(M)** UALCAN database, **(N)** GSE45827. **(O)** MTHFD2 protein expression level in breast cancer and normal breast tissue from the HPA database (antibody, HPA049657). **(P)** Nomogram model integrating MTHFD2 expression and prognostic factors in breast cancer. **(Q)** Calibration curve of the nomogram. **(R)** The receiver operating characteristics curve shows an AUC value of 0.908 (confidence interval: 0.888-0.929) for distinguishing between normal and tumor tissue. ns: *p*≥0.05, *p<0.05, **p<0.01, ***p<0.001, and ****p<0.0001.

**Figure 5:**
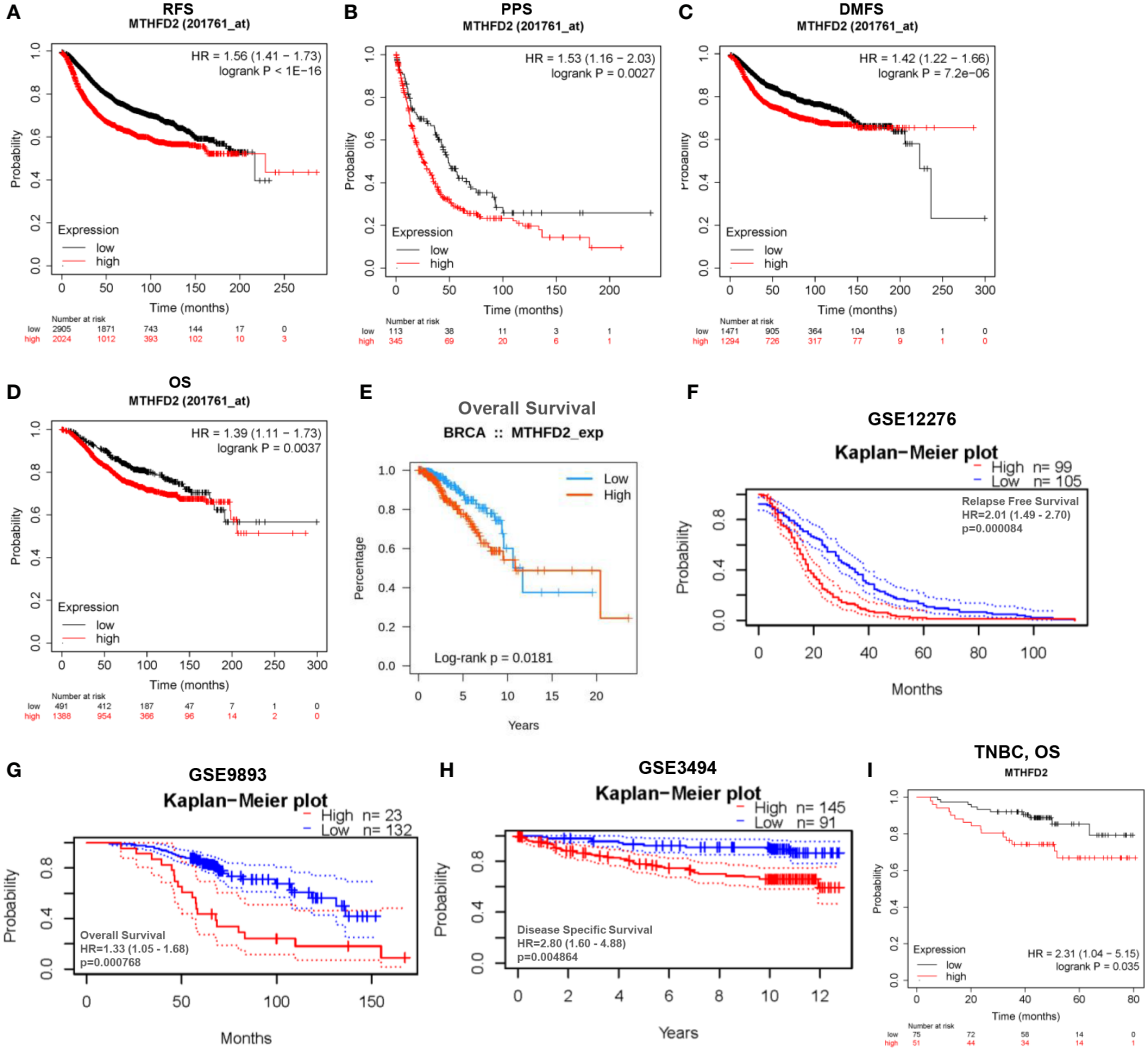
Analysis of patient prognosis in breast cancer. **(A–H)** Relationship between MTHFD2 expression and prognosis in breast cancer patients. **(A–D)** Kaplan-Meier curves were constructed based on the Kaplan-Meier Plotter database. **(A)** RFS, recurrence-free survival; **(B)** PPS, post-progression survival; **(C)** DMFS, distant metastasis-free survival; **(D)** OS, overall survival. **(E)** Survival curves for patients with breast cancer based on data obtained from the TISIDB database. **(F–H)** Kaplan-Meier curves were drawn based on the GEO database with data obtained from the PrognoScan database. **(F)** GSE12276, relapse free survival; **(G)** GSE9893, overall survival; **(H)** GSE3494, disease specific survival. **(I)** Kaplan-Meier curve drawn based on the Kaplan–Meier Plotter database for predicting overall survival in TNBC patients.

Based on data from TCGA and GEO databases, we found mRNA expression levels of MTHFD2 to be prominently up-regulated in breast cancer samples than in normal tissue samples (**Figures 4A–D**). The MTHFD2 expression levels were prominently enhanced in T, N, M, and pathologic stages of tumors compared to normal samples. However, the findings also revealed that higher MTHFD2 expression levels were not associated with more advanced tumor stages (**Figures 4E–H**). Furthermore, higher expression of MTHFD2 was associated with worse T stage and Grade, but not N stage and M stage from the GEO database (**Figures 4I–L**). On subtype analysis of breast cancer based on TCGA and GEO databases, MTHFD2 expression was found to be higher in the basal (TNBC) and human epidermal receptor 2-amplification subtypes than in the luminal subtype (**Figures 4M, N**). Immunohistochemical staining images obtained from the HPA database revealed higher MTHFD2 protein expression in tumors than in normal tissues (**Figure 4O**). A nomogram model was subsequently constructed to estimate the survival probability of breast cancer patients by combining multiple clinical characteristics and MTHFD2 expression levels (**Figure 4P**). A higher score indicated a poorer prognosis in this model. The calibration curve suggested that the nomogram model could reliably predict prognosis in patients with breast cancer (**Figure 4Q**). A receiver operating characteristics curve was constructed to explore the clinical diagnostic value of MTHFD2 in breast cancer and the area under the curve value was 0.908 (confidence interval: 0.888-0.902) (**Figure 4R**). This also confirmed the predictive value of MTHFD2 in breast cancer. Survival curves obtained from various databases demonstrated that higher MTHFD2 expression conferred poorer prognosis with both breast cancer (**Figures 5A–H**) and TNBC (**Figure 5I**).

### Functional enrichment analysis

**Figure 6:**
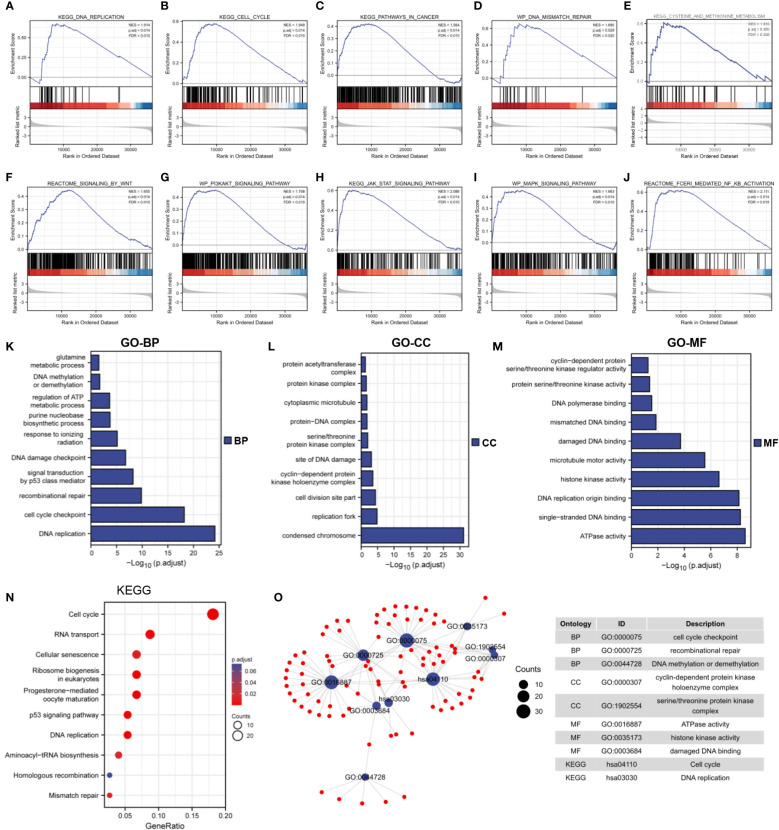
Functional enrichment analysis. **(A–J)** GSEA enrichment analysis identified the biological signature in breast cancer. **(A)** DNA replication, **(B)** cell cycle, **(C)** cancer pathways **(D)** DNA mismatch repair, **(E)** methionine metabolism, **(F)** Wnt signaling, **(G)** PI3K-AKT signaling, **(H)** JAK-STAT signaling, **(I)** MAPK signaling, **(J)** NF-κB signaling. ES, enrichment score; NES, normalized ES; NOM p-Val, normalized p-value; FDR, False Discovery Rates. **(K–M)** GO enrichment analysis of 300 top co-expressed genes in breast cancer. **(K)** biological processes (BP), **(L)** cellular components (CC), **(M)** molecular functions (MF). **(N)** KEGG enrichment analysis of 300 top co-expressed genes in breast cancer. **(O)** Network diagram of GO and KEGG pathway in top 10 tumor-related terms. Ontology, ID, and description of terms are shown in the table.

The underlying biological function and tumor-related signaling pathways of MTHFD2 in breast cancer were predicted by GSEA. The results showed that MTHFD2 may be involved in various intracellular tumor-related biological processes including the cell cycle, DNA mismatch repair, DNA replication, cancer pathways, methionine metabolism (**Figures 6A–E**), and Wnt, MAPK, PI3K-AKT, JAK-STAT, and NF-κB signaling pathway activation (**Figures 6F–J**).

We performed Spearman correlation analysis for MTHFD2 gene expression and screened 300 genes that were most closely related to MTHFD2 (for performing KEGG and GO enrichment analyses). Our results showed that MTHFD2 was closely associated with biological functions such as DNA repair, kinase activity, energy metabolism, cell cycle regulation, and DNA replication (**Figures 6K–N**). On using the screened terms to build an interaction network, we found multiple genes to be enriched for multiple tumor-associated terms (**Figure 6O**).

### Immune infiltration analysis

**Figure 7:**
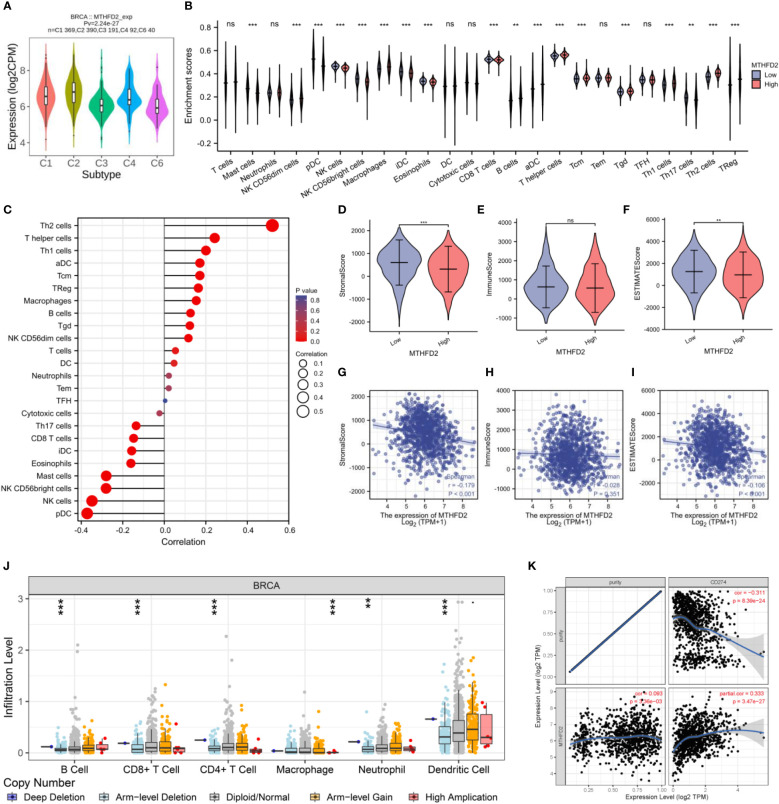
The correlation between MTHFD2 expression and infiltrating immune cells in breast cancer. **(A)** Correlation analysis between MTHFD2 expression and immune subtypes. C1: wound healing, C2: interferon-gamma dominant, C3: inflammatory, C4: lymphocyte depleted, C5: immunologically quiet, C6: TGF-β dominant. **(B)** Analysis of immune cell infiltration in breast cancer patients with high and low MTHFD2 expression. **(C)** Correlation analysis between MTHFD2 expression and immune cell infiltration. **(D–F)** Stromal, immune, and ESTIMATE scores in high and low MTHFD2 expression groups. **(G–I)** Correlation analysis between MTHFD2 expression and stromal, immune, and ESTIMATE scores. **(J)** Correlation analysis between MTHFD2 mutation and immune infiltration. **(K)** Correlation analysis of MTHFD2 expression with immune checkpoints PD-L1 in breast cancer. ns: *p*≥0.05, *p<0.05, **p<0.01, ***p<0.001, ****p<0.0001.

The TISIDB website was used to explore the correlation between MTHFD2 expression and immune subtypes. MTHFD2 expression levels were the highest in the C2 subtype and relatively lower in the C3 and C6 subtypes (**Figure 7A**). Meanwhile, we demonstrated that the group with elevated expression MTHFD2 revealed higher immune cells infiltration levels compared to the lower MTHFD2 expression group, such as B cells, macrophages, aDC, Tgd, Tcm, Treg, and T helper cells (Th1 cells and Th2 cells) (**Figure 7B**). Notably, MTHFD2 expression levels were positively related to the infiltration status of various immune cells, including activated dendritic, T helper (Th1 and Th2), central memory T, and regulatory T cells (**Figure 7C**).

On employing the ESTIMATE algorithm to estimate the impact of MTHFD2 on the TME of breast cancer, the group with high MTHFD2 expression was found to have lower stroma and ESTIMATE scores (but not immune scores). In addition, MTHFD2 expression was negatively related to the stroma and ESTIMATE scores, but not the immune score (**Figures 7D–I**). The relationship between concrete immune markers and MTHFD2 was evaluated using the TIMER and TCGA databases (**Table 1**). The effect of MTHFD2 gene changes on immune infiltration was also investigated, and deletion of MTHFD2 was found to down-regulate the level of immune infiltration (**Figure 7J**). PD-L1 is a crucial immune checkpoint in tumor cells, which could bind to PD-1 on immune cells, resulting in the suppression of immune cells (29). We explore the correlation between the MTHFD2 expression levels and PD-L1 conditions. The result determined that PD-L1 expression levels were positively correlated with the condition of MTHFD2 expression in breast cancer (**Figure 7K**), which meant that PD-L1 inhibitors may be a promising therapeutic approach in breast cancer patients with MTHFD2 expression.

**Table 1:** Correlation analysis between MTHFD2 and immune biomarkers by TIMER and TCGA databases.

Cell type	Gene marker	BRCA TIMER				BRCA TCGA	
		None		Purity		cor	p
		cor	p	cor	p		
B cell	CD19	0.048	0.114	0.105	****	0.028	0.353
	CD79A	0.057	0.058	0.131	****	0.047	0.114
Th1	IFNG	0.198	****	0.264	****	0.180	****
	STAT1	0.345	****	0.374	****	0.347	***
	STAT4	0.073	*	0.151	****	0.051	0.092
	TBX21	0.080	**	0.164	****	0.069	0.022
Th2	GATA3	-0.243	****	-0.284	****	-0.198	****
	STAT6	-0.209	****	-0.188	****	-0.156	****
	STAT5A	-0.191	****	-0.168	****	-0.158	****
	IL13	0.114	****	0.131	****	0.038	0.209
CD8+T cell	CD8A	0.091	**	0.175	****	0.104	****
	CD8B	0.077	**	0.157	****	0.088	**
T cell	CD3D	0.033	0.271	0.110	****	0.038	0.203
	CD3E	0.048	0.108	0.132	****	0.050	0.098
	CD2	0.103	****	0.190	****	0.096	***
M1 Macrophages	PTGS2	0.055	0.070	0.112	****	0.044	0.145
	IRF5	-0.035	0.243	-0.022	0.057	-0.042	0.164
	NOS2	0.046	0.129	0.049	*	0.083	**
M2 Macrophages	MS4A4A	0.175	****	0.237	****	0.156	****
	VSIG4	0.074	*	0.112	****	0.083	**
	CD163	0.243	****	0.294	****	0.217	****
Dendritic cell	ITGAX	0.065	0.031	0.112	****	0.029	0.339
	NRP1	0.032	0.283	0.077	*	0.030	0.316
	CD1C	-0.141	****	-0.107	****	-0.133	****
NK cell	KIR2DS4	0.098	***	0.142	****	0.081	**
	KIR3DL3	0.114	****	0.101	***	0.088	**
	KIR3DL2	0.112	****	0.168	****	0.145	****
	KIR3DL1	0.082	**	0.154	****	0.134	****
Neutrophils	CCR7	0.062	*	0.139	****	0.059	*
	ITGAM	0.013	0.677	0.034	0.288	0.029	0.336
	CEACAM8	0.035	0.242	0.028	0.385	-0.033	0.270
Tfh	BCL6	-0.127	****	-0.117	****	-0.122	****
	IL21	0.195	****	0.234	****	0.241	****
Th17	STAT3	0.061	*	0.07	*	0.104	****
	IL17A	0.117	****	0.141	****	0.100	****
Treg	FOXP3	0.225	****	0.290	****	0.196	****
	CCR8	0.317	****	0.363	****	0.294	****
	STAT5B	-0.120	****	-0.109	****	-0.101	****
	TGFB1	-0.216	****	-0.198	****	-0.174	****
Mast cell	TPSB2	-0.323	****	-0.311	****	-0.293	****
	TPSAB1	-0.315	****	-0.301	****	-0.287	****
	CPA3	-0.183	****	-0.161	****	-0.184	****
	MS4A2	-0.174	****	-0.155	****	-0.192	****
	HDC	-0.222	****	-0.202	****	-0.232	****

*p<0.05, **p<0.01, ***p<0.001, and ****p<0.0001.

### Identification of MTHFD2 expression level

**Figure 8:**
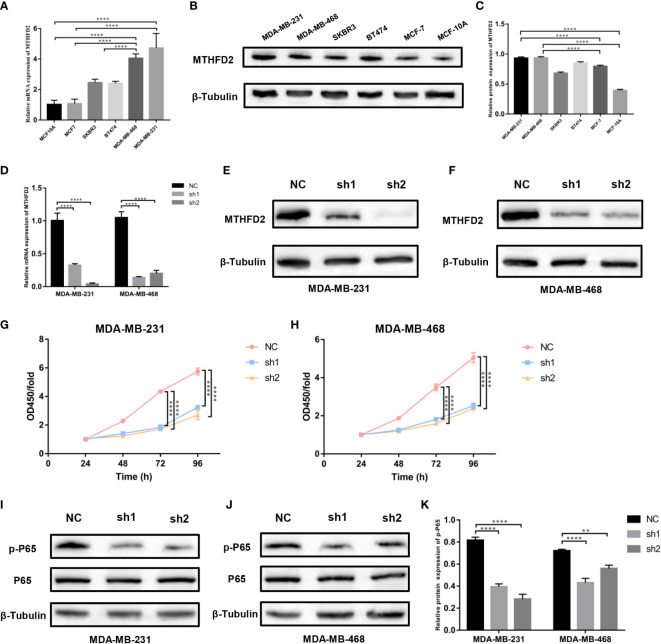
Expression levels of MTHFD2 in various breast cancer cell lines, construction of MTHFD2 knockdown cell lines, and cell proliferation assay. **(A)** Determination of MTHFD2 mRNA expression levels in normal breast cell lines (MCF-10A) and breast cancer cell lines (BT474, MDA-MB-468, MCF7, SKBR3, and MDA-MB-231). **(B, C)** Determination of MTHFD2 protein expression levels in normal breast cell lines (MCF-10A) and breast cancer cell lines (BT474, MDA-MB-468, MCF7, SKBR3, and MDA-MB-231). **(D)** RT-qPCR was used to verify the knockdown effect of MTHFD2 mRNA in MDA-MB-231 and MDA-MB-468 cell lines. **(E, F)** Western blot assay verified the knockdown effect of MTHFD2 protein in MDA-MB-231 and MDA-MB-468 cell lines. **(G, H)** CCK8 assays were used to detect the effect of MTHFD2 knockdown on the viability of MDA-MB-231 and MDA-MB-468 cell lines. **(I-K)** Western blot was used to verify the expression of p-P65 and P65 in MTHFD2-knockdown MDA-MB-231 and MDA-MB-468 cell lines. ns: *p*≥0.05, *p<0.05, **p<0.01, ***p<0.001, ****p<0.0001.

RT-qPCR and Western blot assays were performed to detect MTHFD2 mRNA and protein expression levels in multiple human breast cancer cell lines (MDA-MB-468, MDA-MB-231, MCF7, SKBR3, and BT474) and the normal human breast cell line (MCF10A). We confirmed MTHFD2 expression levels to be markedly up-regulated in breast cancer cells. TNBC cells (MDA-MB-231 and MDA-MB-468) demonstrated higher MTHFD2 expression than cells of other breast cancer subtypes (**Figures 8A–C**). This was consistent with the findings from the previous bioinformatics analysis.

### The knockdown of MTHFD2 in TNBC cells

We selected TNBC cells (MDA-MB-231 and MDA-MB-468) for the construction of MTHFD2-knockdown cells. The effects of knockdown were identified using RT-qPCR and Western blot assays. On targeting the MTHFD2 short hairpin RNA that was transfected in the TNBC cells, the cells demonstrated prominent downregulation of MTHFD2 mRNA and protein expression (compared to the control group) (**Figures 8D–F**). This indicated the successful establishment of MTHFD2-knockdown cells.

### Effects of MTHFD2 on proliferation

The Cell Counting Kit-8 assay detected differences in cell viability between the control and MTHFD2-knockdown TNBC cell groups. The latter demonstrated a lower optical density value than the former after cultivation for 48h and this effect was most prominent at 96 hours. This suggested significant inhibition of cell proliferation in the TNBC MTHFD2-knockdown cell lines (**Figures 8G, H**). The potential mechanisms by which MTHFD2 knockdown suppressed tumor cell proliferation and increased apoptosis were also investigated. The Western blot assay demonstrated inhibition of NF-κB signaling pathway activation along with down-regulation of MTHFD2 (**Figures 8I–K**).

### Effects of MTHFD2 on apoptosis

**Figure 9:**
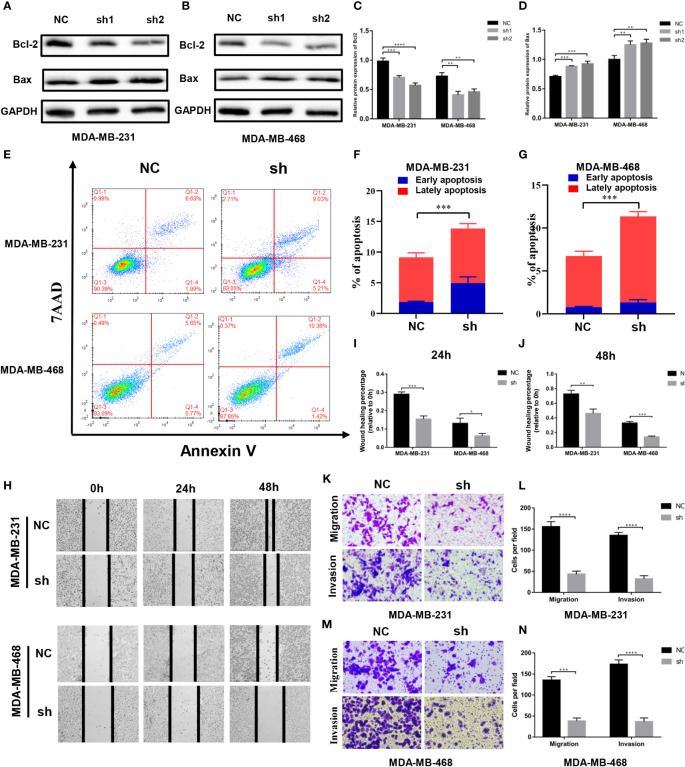
Effect of MTHFD2 knockdown on apoptosis, migration, and invasion in TNBC cell. **(A–D)** Western blot assays were used to verify the expression of Bcl2 and Bax in MTHFD2-knockdown MDA-MB-231 and MDA-MB-468 cell lines. **(E–G)** The effect of MTHFD2 knockdown on apoptosis in MDA-MB-231 and MDA-MB-468 cell lines (measured by flow cytometry). **(H–J)** Effect of MTHFD2 knockdown on migration in MDA-MB-231 and MDA-MB-468 cell lines (measured by wound scratch assay). **(K–N)** Effect of MTHFD2 knockdown on migration and invasion in MDA-MB-231 and MDA-MB-468 cell lines (measured by transwell assay). (ns: *p*≥0.05, *p<0.05, **p<0.01, ***p<0.001, ****p<0.0001).

We found the expression of Bcl2 and Bax proteins to be down- and up-regulated, respectively, with the knockdown of MTHFD2 (**Figures 9A–D**). Therefore, we speculate that MTHFD2 may be associated with apoptosis in TNBC cells. Flow cytometry was subsequently used to examine the apoptosis status in MTHFD2-knockdown TNBC cells. The results suggested that the knockdown of MTHFD2 could increase the overall apoptosis rate in these cells (**Figures 9E–G**).

### Effects of MTHFD2 on migration and invasion

Cell wound scratch experiments were performed to investigate the effect of migration in MTHFD2-knockdown TNBC cells. The MTHFD2-knockdown cells were found to possess a lower healing rate than the control cells (**Figures 9H–J**). The effects of MTHFD2 on migration and invasion of TNBC cells were determined using transwell assays. The results demonstrated that the proportion of cells that passed through the upper chamber was lower in the MTHFD2-knockdown group than in the control group (**Figures 9K–N**). This result was consistent with findings from previous experiments.

### MTHFD2 is a possible inducer of ferroptosis

**Figure 10:**
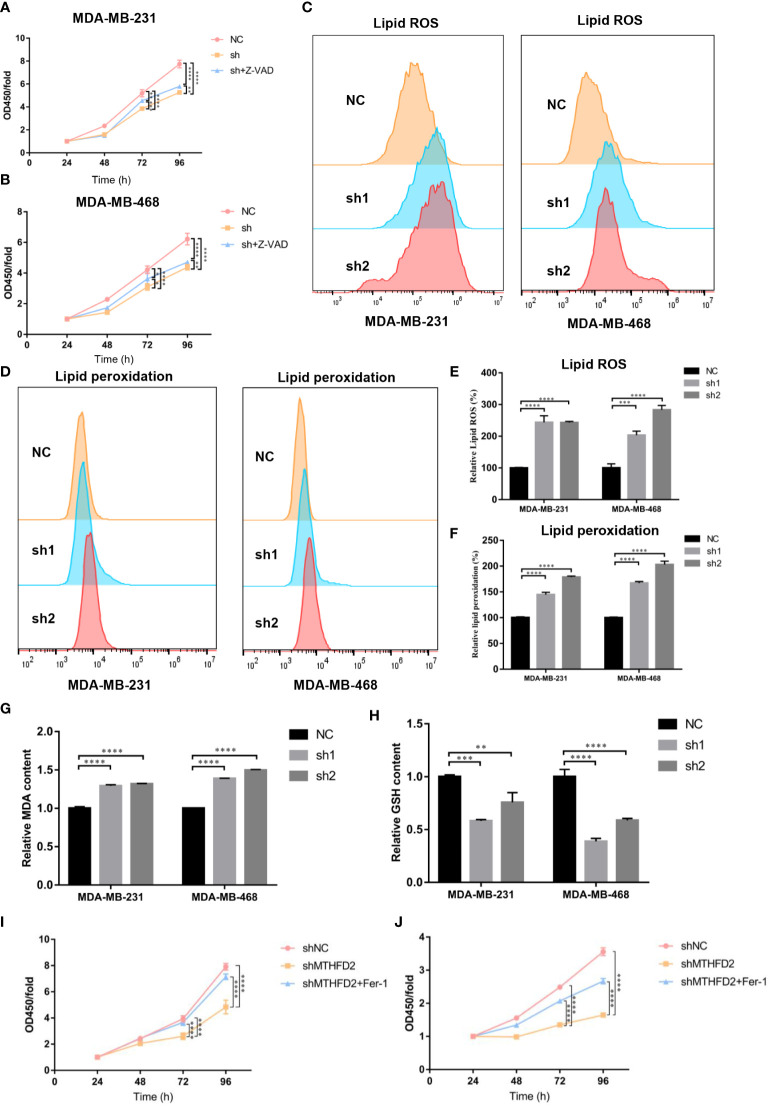
Effect of MTHFD2 knockdown on ferroptosis in TNBC cells. **(A, B)** CCK8 assays were used to measure the effect of MTHFD2 knockdown in MDA-MB-231 and MDA-MB-468 cell lines with Z-VAD-FMK (20 µm/L). **(C, D)** Lipid ROS and lipid peroxidation content in MDA-MB-231 and MDA-MB-468 cell lines (detected by flow cytometry). **(E, F)** Statistical results of lipid ROS and lipid peroxidation content (measured by flow cytometry). **(G)** Effect of MTHFD2 knockdown on MDA in MDA-MB-231 and MDA-MB-468 cell lines. **(H)** Effect of MTHFD2 knockdown on GSH in MDA-MB-231 and MDA-MB-468 cell lines. **(I, J)** CCK8 as were says used to measure the effect of MTHFD2 knockdown in MDA-MB-231 and MDA-MB-468 cell lines with ferrostatin-1 (1 µm/L). ns: *p*≥0.05, *p<0.05, **p<0.01, ***p<0.001, ****p<0.0001.

Previous experiments have confirmed that the knockdown of MTHFD2 may prominently suppress proliferation ability and increase the apoptotic fraction in TNBC cells. Therefore, a pan-caspase inhibitor, Z-VAD-FMK, was used to explore the effect of MTHFD2 on the regulation of cell apoptosis and proliferation in MTHFD2-knockdown cells. Similar to the results of the apoptosis assay, Z-VAD-FMK inhibited the apoptosis caused by MTHFD2 knockdown. However, its inhibitory ability remained limited (**Figures 10A, B**). The ability of Z-VAD-FMK to promote apoptosis did not match the ability to inhibit proliferation. Hence, we speculated that ferroptosis may be involved in MTHFD2-related regulation of proliferation in TNBC cells in combination w to cellular antioxidant biological functions.

Flow cytometry was used to detect intracellular ROS and lipid peroxidation to validate intracellular oxidation levels in the TNBC cell line. Intracellular ROS and lipid peroxidation contents in MTHFD2-knockout cells were found to be more prominently elevated than in the control group (**Figures 10C–F**). MDA is formed by lipid peroxidation in cells (30), and GSH is a significant intracellular antioxidant. We examined the intracellular oxidation levels by MDA and GSH content. The results confirmed that the MDA was significantly enhanced and the GSH declined in the MTHFD2-knockdown cell group in TNBC cells (**Figures 10G, H**). Ferrostatin-1 (Fer-1), a ferroptosis inhibitor, was added to investigate the role of ferroptosis in MTHFD2-related regulation of tumor cell proliferation in TNBC cell lines. The Cell Counting Kit-8 assay results showed that ferrostatin-1 could promote TNBC cell proliferation in the MTHFD2-knockdown group (**Figures 10I, J**). Ferrostatin-1 suppressed MTHFD2-knockdown cell proliferation more significantly than Z-VAD-FMK.

### Regulatory mechanism of MTHFD2-induced ferroptosis

**Figure 11:**
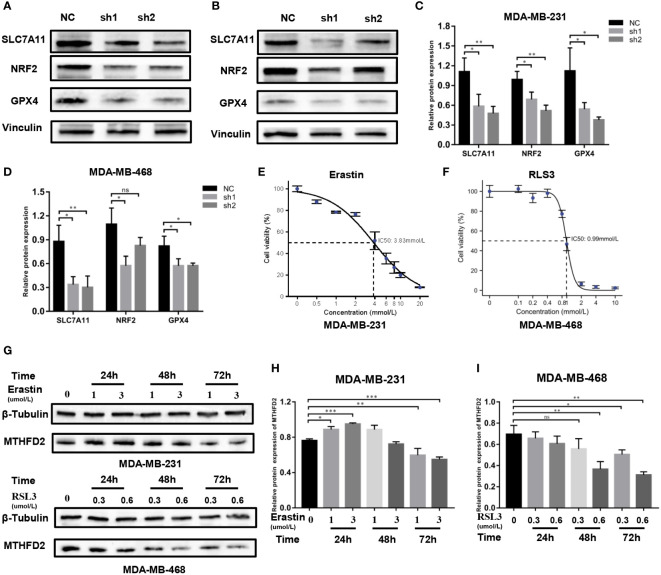
Regulatory mechanisms of MTHFD2 in ferroptosis. **(A-D)** Western blot assays were used to detect the expression of ferroptosis-related proteins in MDA-MB-231 and MDA-MB-468 cell lines. **(E)** IC50 of erastin in the MDA-MB-231 cell line (detected by CCK8 assay); the IC50 is approximately 3.83 µm/L. **(F)** IC50 of RLS3 in the MDA-MB-468 cell line (detected by CCK8 assay); the IC50 is approximately 0.99um/L. **(G-I)** Expression of MTHFD2 in MDA-MB-231 and MDA-MB-468 after erastin or RLS3 treatment (detected by Western blot assays). ns: *p*≥0.05, *p<0.05, **p<0.01, ***p<0.001, ****p<0.0001.

The possible regulatory mechanism of MTHFD2 in ferroptosis was investigated using the Western blot assay. Our results demonstrated the expression levels of classical ferroptosis regulatory proteins including GPX4, NRF2, and SLC7A11, to be prominently down-regulated in MTHFD2-knockdown TNBC cells (**Figures 11A–D**).

Erastin and RSL3 are both inducers of ferroptosis. The half maximal inhibitory concentration (IC50) of erastin in MDA-MB-231 cells was 3.83 umol/L, while that of RSL3 in MDA-MB-468 cells was 0.9 µmol/L (**Figures 11E, F**). Appropriate concentrations were selected to stimulate TNBC cells based on the determined IC50 data. Western blot assays were then performed to identify the MTHFD2 protein expression status. The result showed that in MDA-MB-231 cells the expression of MTHFD2 protein was slightly up-regulated and then significantly down-regulated as time extended, rather than the concentration of Erastin. The downregulation effect was most significant at 72 hours (**Figures 11G, H**). The levels of MTHFD2 protein expression declined prominently with an increase in RSL3 addition time and concentrations (**Figures 11G, I**).

## Discussion

MTHFD2 is an important molecule encoded by the nucleus. It is involved in folate metabolism and the synthesis of one-carbon units in mitochondria (5). The regulation of the synthesis of one-carbon, which is one of the main materials for DNA synthesis, could be involved in tumor-related biological processes (31). MTHFD2 is also involved in the regulation of intracellular NADPH and GSH and maintains intracellular redox balance due to its NADPH-dependence (8). In this study, bioinformatics analysis demonstrated that MTHFD2 is highly expressed in multiple tumors and that its expression level correlates with the progression of malignancy. These tumor-related biological characteristics may be attributed to the MTHFD2-related promotion of intracellular DNA synthesis, which may reduce intracellular oxidation levels and cell death. This may in turn increase cell proliferation ability and resistance to the external environment.

Li et al. found that the STAT3 and epithelial-mesenchymal transition (EMT) signaling pathways were involved in the MTHFD2 regulation process on the proliferation and metastasis of ovarian cancer (15). This suggests that MTHFD2-induced activation of intracellular signaling pathways could also be involved in the malignant proliferation of tumors. The enrichment analysis results identified the MAPK, WNT, PI3K-AKT, JAK-STAT, and NF-κB signaling pathways to be potentially involved in the regulation of MTHFD2-induced proliferation of breast cancer. Findings from the Western blot assays suggested that MTHFD2 knockdown could suppress NF-κB signaling pathway activation and transduction. In this context, MTHFD2 also regulates apoptosis-related genes, including Bcl2 and Bax, to affect apoptosis in breast cancer. We found that in addition to being highly expressed in breast cancer (especially in TNBC), MTHFD2 modulated the proliferative, migratory, invasive, and apoptotic ability of TNBC cells. MTHFD2 may therefore be a crucial biomarker for predicting patient prognosis and a potential therapeutic target in TNBC.

Immune cells are one of the major components of the TME and have the ability to affect tumor function and tumor therapy outcomes (32). Previous studies on ovarian carcinoma have shown that patients with tumor T-cell infiltration had significantly longer survival than those without (33, 34). Innate immune cells, such as dendritic cells, neutrophils, natural killer cells, myeloid-derived suppressor cells, and macrophages in the TME could also regulate tumor immune function by secreting cytokines (35). The interactions between various immune cells in the TME collectively regulate the biological functions of tumor cells. In our study, we explored the correlation between MTHFD2 expression levels and the TME (particularly immune cell infiltration) in breast cancer. The results confirmed the MTHFD2 expression levels to be positively associated with tumor-infiltrating immune cells. The deletion of MTHFD2 copy numbers was the most critical factor affecting immune infiltration in breast cancer. The findings also suggested that MTHFD2 expression correlated significantly with the stromal and estimate scores, but not with the immune score.

In recent years, immunotherapy, mainly represented by immune checkpoint inhibitors, including PD-1/PD-L1 and CTLA-4 inhibitors, has been widely applied in the treatment of various cancers with satisfactory results, which has become an indelible milestone in tumor immunotherapy (36, 37). Shang et al. demonstrated that MTHFD2 could up-regulate PD-L1 expression, leading to immune cell evasion in tumors. Mechanistically, MTHFD2 promotes cMYC O-GlcNAcylation by promoting folate metabolism to generate the uridine-related metabolite UDP-GlcNAc, which ultimately increases cMYC stability and PD-L1 transcription (38). The correlation between the expression of MTHFD2 and multiple immune checkpoint molecules in breast cancer was also evaluated in this study. Correlation analysis revealed that the levels of PD-1/PD-L1 and CTLA-4 expression were positively associated with that of MTHFD2. In this context, findings from studies suggest that the expression level of immune checkpoints strongly affects immunotherapy efficacy (39). Hence, immune checkpoint inhibitors are promising therapies for MTHFD2-mediated triple-negative breast cancer (40).

The regulated cell death (RCD) processes, including necroptosis, apoptosis, ferroptosis, and pyroptosis et al., were the special death process that is different from accidental cell death, which may be controlled by various specific signal transduction pathways, drugs, and genetic intervention (41). By regulating the RCD process, it is more accurate to eliminate as many tumor cells as possible while reducing the damage to normal cells. The mode of RCD associated with metallic elements such as iron, manganese, and copper, is a novel approach, which has its own unique molecular regulation mechanism (42–45). The classic induction process of ferroptosis involves membrane rupture and eventual cell death owing to the accumulation of lipid peroxides on the cell membrane in lethal concentrations, which exceed the buffering capacity of the intracellular defense system (43). The main molecular pathways of ferroptosis involve exogenous and endogenous regulatory processes. The interaction of these two pathways together regulates the occurrence of ferroptosis. The exogenous pathway of ferroptosis mainly involves suppression of the cystine/glutamate transporter complex or activation of iron transporters such as serotransferrin or lactotransferrin. The endogenous regulatory pathway blocks cellular antioxidant enzymes including GSH peroxidase 4 (GPX4) to regulate the occurrence of ferroptosis (46). During ferroptosis, cells demonstrate the accumulation of considerable quantities of polyunsaturated fatty acid-containing phospholipids, ROS, and iron, and the inactivation of intracellular ferroptosis defense systems including GPX4-GSH, FSP1-CoQH2, DHODH-CoQH2, and DHODH-CoQH2 (47).

In our experiments, we found that the intracellular ROS, lipid peroxidation, and MDA levels were significantly up-regulated in MTHFD2 knockdown cells compared to control cells. Meanwhile, GSH, an essential antioxidant belonging to the GPX-GSH system in the cell, decreased in the TNBC cells with the MTHFD2 down-regulation. Moreover, the expression of several crucial ferroptosis regulatory molecules including GPX4, NRF2, and SLC7A11 was also suppressed in MTHFD2-knockdown TNBC cells. Therefore, we speculate that MTHFD2 knockdown cells undergo ferroptosis, which ultimately leads to a reduction in cell proliferation and migration.

Ferroptosis has also been reported to be potentially tumor-biologically modulated and plays a crucial role in improving the efficacy of cancer therapy. Jiang et al. found that p53 decreased cellular GSH content and sensitized the cells to ferroptosis by inhibiting SLC7A11 expression and cystine uptake (22). Zhang et al. demonstrated that BAP1 could inhibit the expression of SLC7A11 by decreasing the occupancy of the H2Aub promoter, resulting in inhibited intracellular cystine uptake and increased lipid peroxidation levels (48). In hepatocellular carcinoma cells, the NF-κB signaling pathway has been found to be involved in the ferroptosis regulation process (49).

The unique metabolic changes, mutations of tumor-related genes, and imbalance of redox levels collectively impair the function of the ferroptosis defense system in tumor cells and inhibit the occurrence of ferroptosis (50, 51). These specific biological functions make ferroptosis a natural barrier to the process of tumor occurrence, metastasis, and therapy resistance (52). The combination of ferroptosis inducers with conventional therapies has shown great potential in cancer treatment (53). Immunotherapy has been found to sensitize tumors to radiotherapy by inducing ferroptosis in tumor cells. In this context, Lang et al. reported that immunotherapy-activated CD8+ T cells and radiotherapy-activated interferon γ could synergistically suppress SLC7A11 expression, which reduced cystine uptake by tumor cells, enhanced lipid oxidation and ferroptosis of tumors, and inhibited tumor proliferation (54). Therefore, ferroptosis is a potential regulator that improves therapeutic efficacy in cancer.

In our study, we demonstrated that MTHFD2 knockdown could indeed affect the proliferation of TNBC cells through ferroptosis. We also confirmed that MTHFD2 was involved in the regulation of the NF-κB signaling pathway. However, it remains unclear whether MTHFD2 can regulate ferroptosis by the NF-κB signaling pathway and its specific regulatory network in TNBC cells is also unknown. In addition, we found that MTHFD2 can regulate the expression of various ferroptosis-related genes, but its specific regulatory mechanism requires additional investigation.

## Conclusion

The findings from this study suggest that MTHFD2 could be a crucial molecular prognostic biomarker and a novel therapeutic target in TNBC. The findings also indicate that MTHFD2 is a potential ferroptosis regulatory gene in TNBC.

## Data availability statement

The datasets presented in this study can be found in online repositories. The names of the repository/repositories and accession number(s) can be found in the article/**Supplementary Material**.

## Author contributions

The study was conceived and designed by HX. HZ performed the most statistical analysis and experiment and wrote the manuscript. SZ, HTZ, RL and XX participated in collecting literature and helped to revise the manuscript. All authors contributed to the article and approved the submitted version.
