# Supplementary material for: Identification of MTHFD2 as a prognostic biomarker and ferroptosis regulator in triple-negative breast cancer
Source: Front Oncol. 2023 Jan 16;13:1098357. doi: 10.3389/fonc.2023.1098357 (PMC9885267; doi:10.3389/fonc.2023.1098357)
Supplement: Supplementary file 1 [file Table_1.docx]

| Name | Web address |
| --- | --- |
| The Cancer Genome Atlas (TCGA) | https://portal.gdc.cancer.gov/ |
| UCSC Xena | http://xena.ucsc.edu/ |
| Genotype Tissue-Expression (GTEX) | https://www.gtexportal.org/ |
| Gene Expression Omnibus (GEO) | https://www.ncbi.nlm.nih.gov/geo/ |
| Xiantao scholarship | https://www.xiantao.love |
| UALCAN database | http://ualcan.path.uab.edu |
| Human Protein Atlas (HPA) | https://www.proteinatlas.org/ |
| Kaplan–Meier Plotter | https://kmplot.com/analysis |
| TISIDB | http://cis.hku.hk/TISIDB/ |
| PrognoScan | http://dna00.bio.kyutech.ac.jp/PrognoScan/index.html |
| cBioPortal | http://www.cbioportal.org |
| Gene Set Cancer Analysis (GSCA) | http://bioinfo.life.hust.edu.cn |
| TIMER | https://cistrome.shinyapps.io/timer/ |
