# Supplementary material for: Identification of MTHFD2 as a prognostic biomarker and ferroptosis regulator in triple-negative breast cancer
Source: Front Oncol. 2023 Jan 16;13:1098357. doi: 10.3389/fonc.2023.1098357 (PMC9885267; doi:10.3389/fonc.2023.1098357)
Supplement: Supplementary file 2 [file Table_2.docx]

| Gene | Sequences |
| --- | --- |
| MTHFD2 forward | 5’-GATCCTGGTTGGCGAGAATCC-3’ |
| MTHFD2 reverse | 5’- TCTGGAAGAGGCAACTG-3’ |
| GAPDH forward | 5’-GGAGCGAGATCCCTCCAAAAT-3’ |
| GAPDH reverse | 5’-GGCTGTTGTCATACTTCTCATGG-3 |
