# Supplementary material for: Identification of MTHFD2 as a prognostic biomarker and ferroptosis regulator in triple-negative breast cancer
Source: Front Oncol. 2023 Jan 16;13:1098357. doi: 10.3389/fonc.2023.1098357 (PMC9885267; doi:10.3389/fonc.2023.1098357)
Supplement: Supplementary file 3 [file Table_3.docx]

| Antibody | Manufacturer | Product code | Dilution rate |
| --- | --- | --- | --- |
| anti-GAPDH | Proteintech | 60004-1-Ig | 1:100000 |
| anti-β-Tubulin | Proteintech | 10068-1-AP | 1:2000 |
| anti-Vinculin | Proteintech | 66305-1-Ig | 1:5000 |
| anti-MTHFD2 | Proteintech | 12270-1-AP | 1:1000 |
| anti-Bcl-2 | Abcam | ab182858 | 1:2000 |
| anti-Bax | Abcam | ab182733 | 1:2000 |
| anti-p65 | CST | #8242 | 1:1000 |
| anti-Phospho-p65 (Ser536) | CST | #3033 | 1:1000 |
| anti-SLC7A11 | Abcam | ab175186 | 1:2000 |
| anti-GPX4 | Abcam | ab125066 | 1:2000 |
| anti-NRF2 | Abcam | ab137550 | 1:1000 |
| anti-Rabbit IgG | Proteintech | SA00001-2 | 1:6000 |
| anti-Mouse IgG | Proteintech | SA00001-1 | 1:6000 |
